# Supplementary material for: Validation of a QTL on Chromosome 1DS Showing a Major Effect on Salt Tolerance in Winter Wheat
Source: Int J Mol Sci. 2022 Nov 8;23(22):13745. doi: 10.3390/ijms232213745 (PMC9691212; doi:10.3390/ijms232213745)
Supplement: Supplementary file 1 [file ijms-23-13745-s001.zip › ijms-1964779-supplementary.pdf]

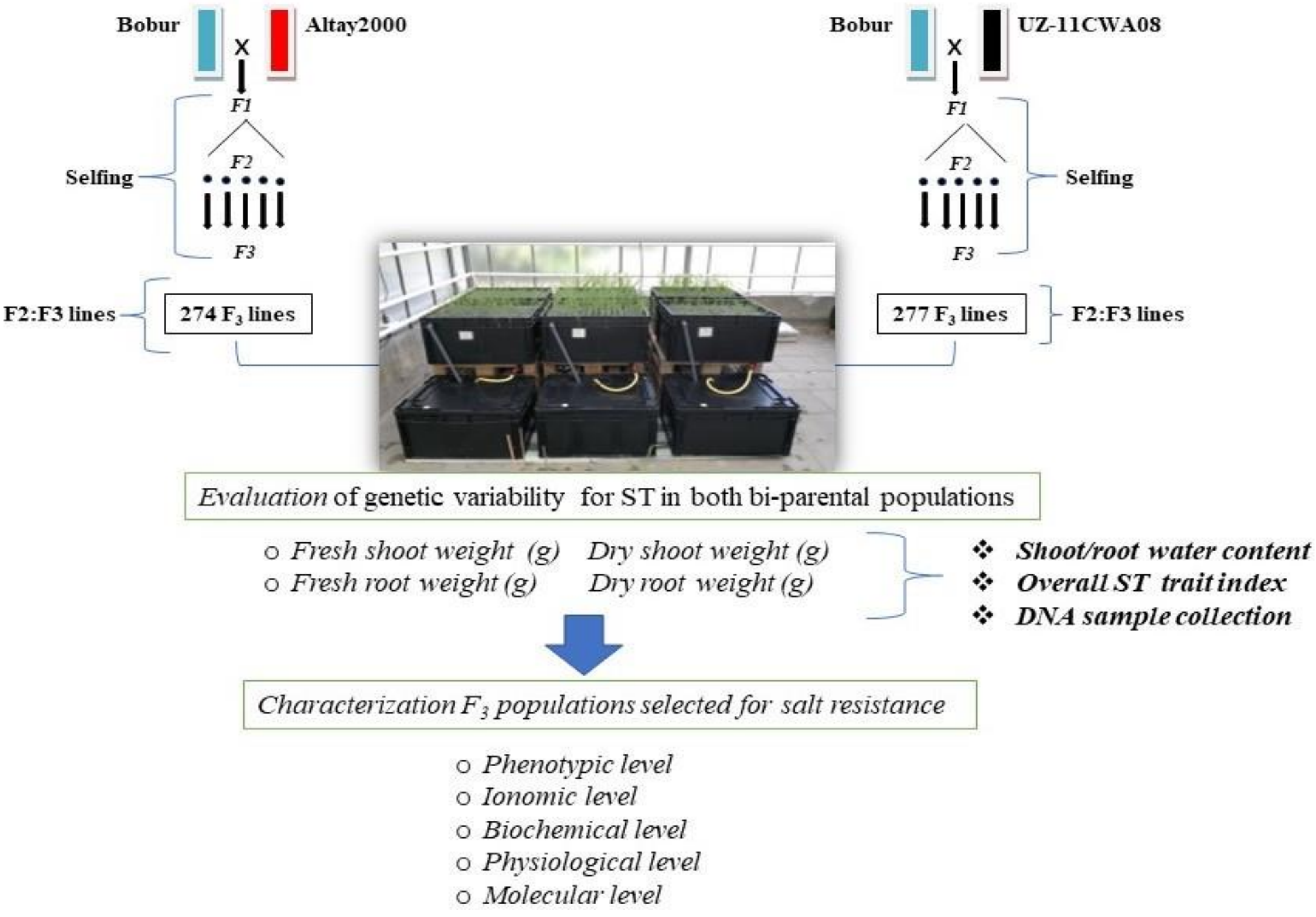

Figure S1 Establishment of the F_3 lines Bobur*Altay2000 and Bobur* UZ-11CWA08 and Schematic framework of the work steps**.**

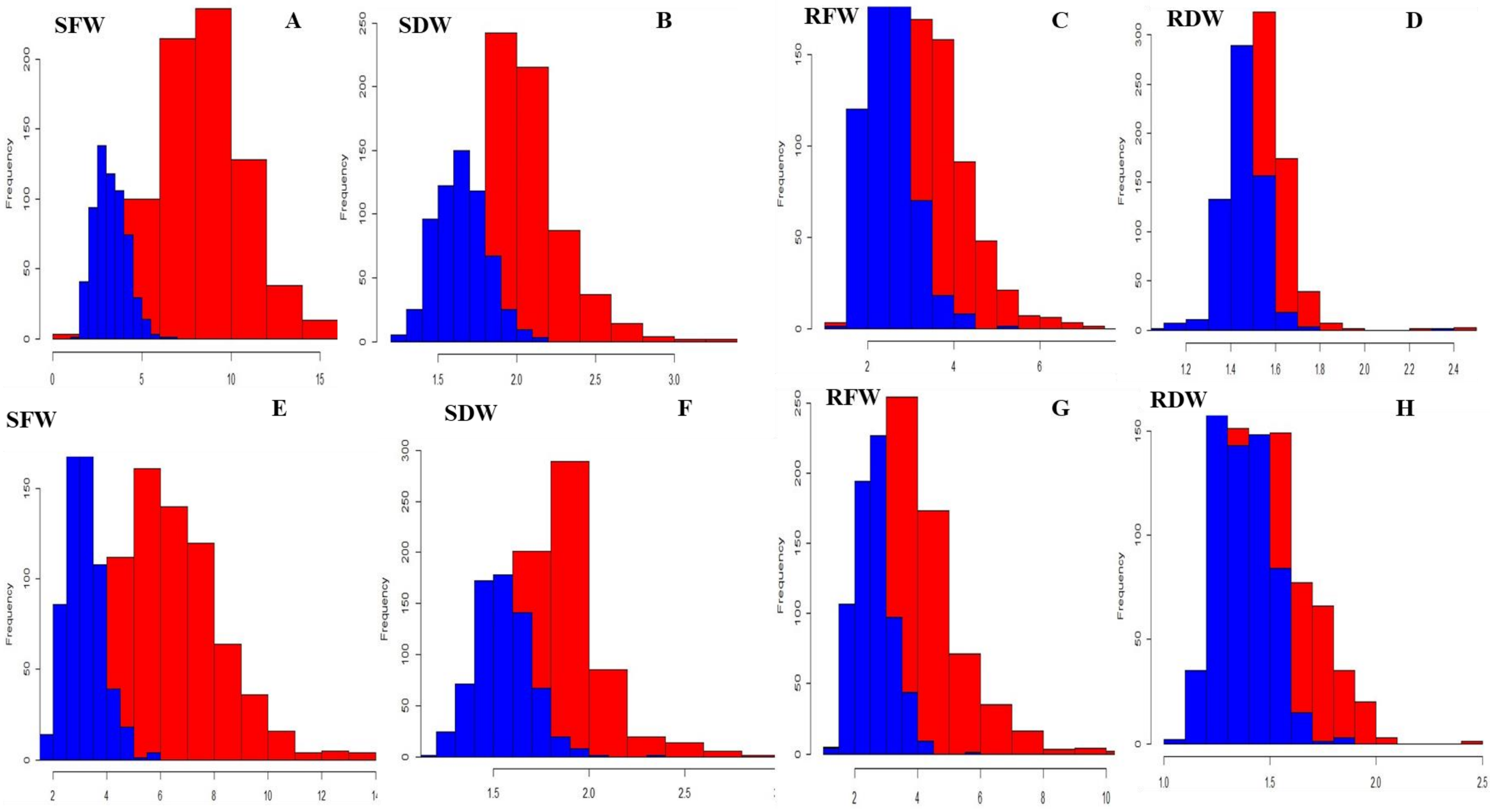

Figure S2 (A-D) Histogram analysis of the measured traits under non saline (Red) and saline (Blue) conditions of the F_3 lines Bobur*Altay2000 and **(E-H)** of the F_3 lines of cross Bobur*UZ-11CWA08.**SFW** shoot fresh weight**, SDW** shoot dry weight**, RFW** root fresh weight**, RDW** root dry weight.

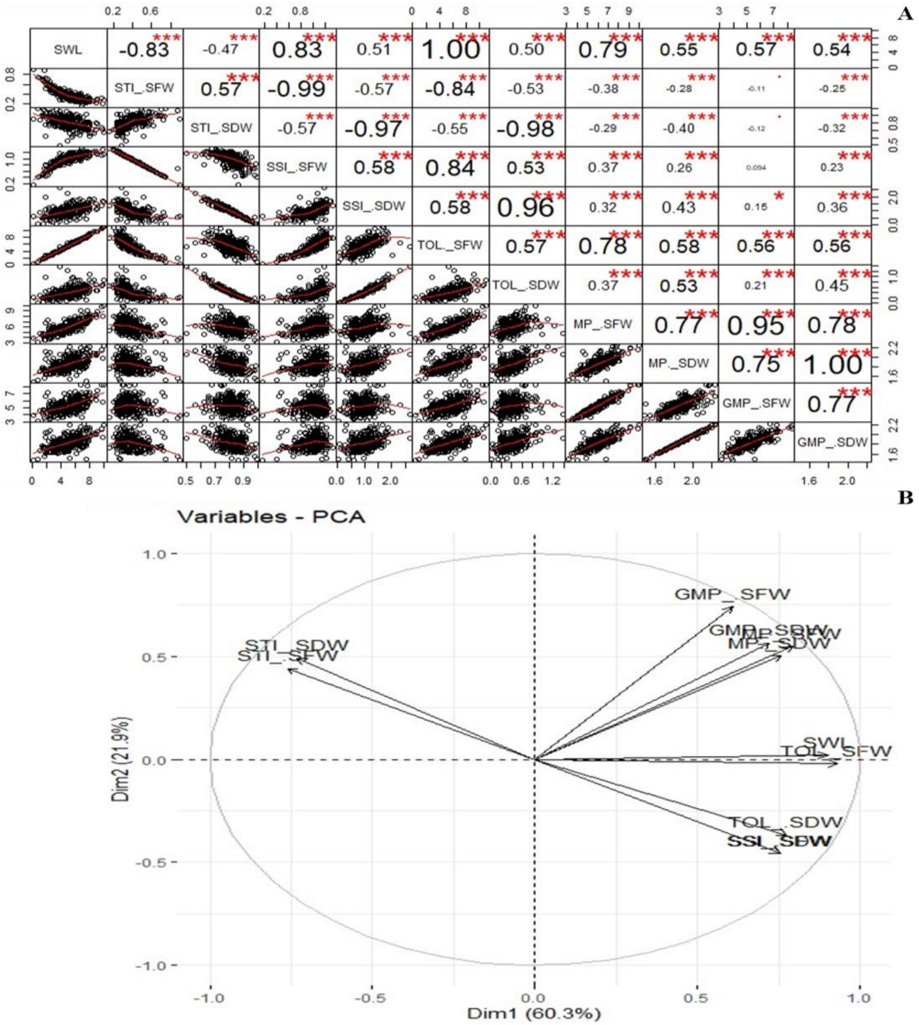

Figure S3. (A) Simple correlation coefficients between SWL and some salt tolerance indices of F_3 lines of cross Bobur*Altay2000. **(B)** Graphic display biplot for some salt tolerance indices and SWL based on 274 F_3 lines of cross Bobur*Altay2000. Note: F-values are shown; significance levels *p*: * $p \leq 0.05$; *** $p \leq 0.001$. ns, not significant.

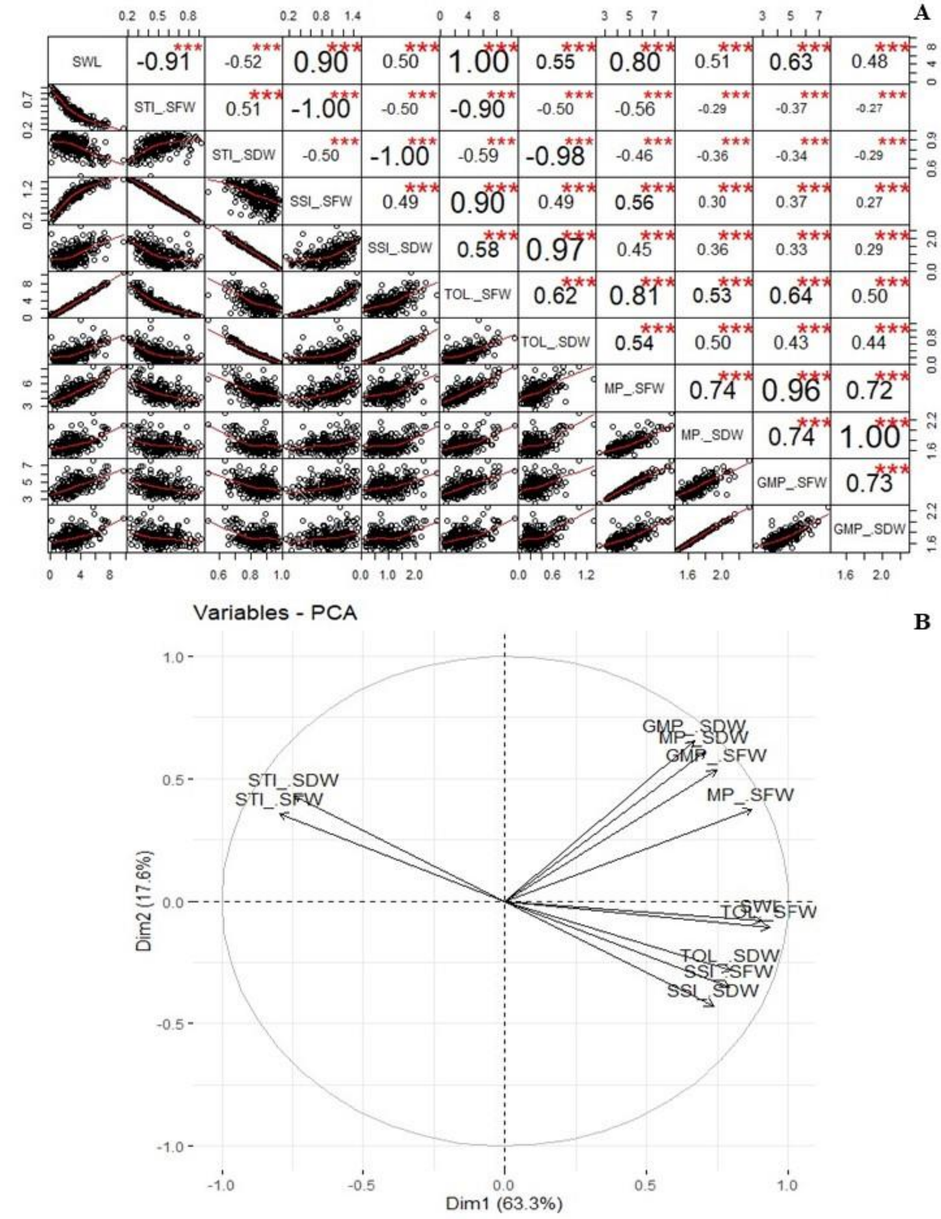
A
B
SWL
STI_SFW
STI_SDW
SSI_SFW
SSI_SDW
TOL_SFW
TOL_SDW
MP_SFW
MP_SDW
GMP_SFW
GMP_SDW
Variables - PCA
Dim1 (63.3%)
Dim2 (17.6%)

Figure S4. (A) Simple correlation coefficients between SWL and some salt tolerance indices of F_3 lines of cross Bobur*UZ-11CWA08. **(B)** Graphic display biplot for some salt tolerance indices and SWL based on 277 F_3 lines of cross Bobur*UZ-11CWA08. Note: F-values are shown; significance levels *p*: * $p \leq 0.05$; *** $p \leq 0.001$.

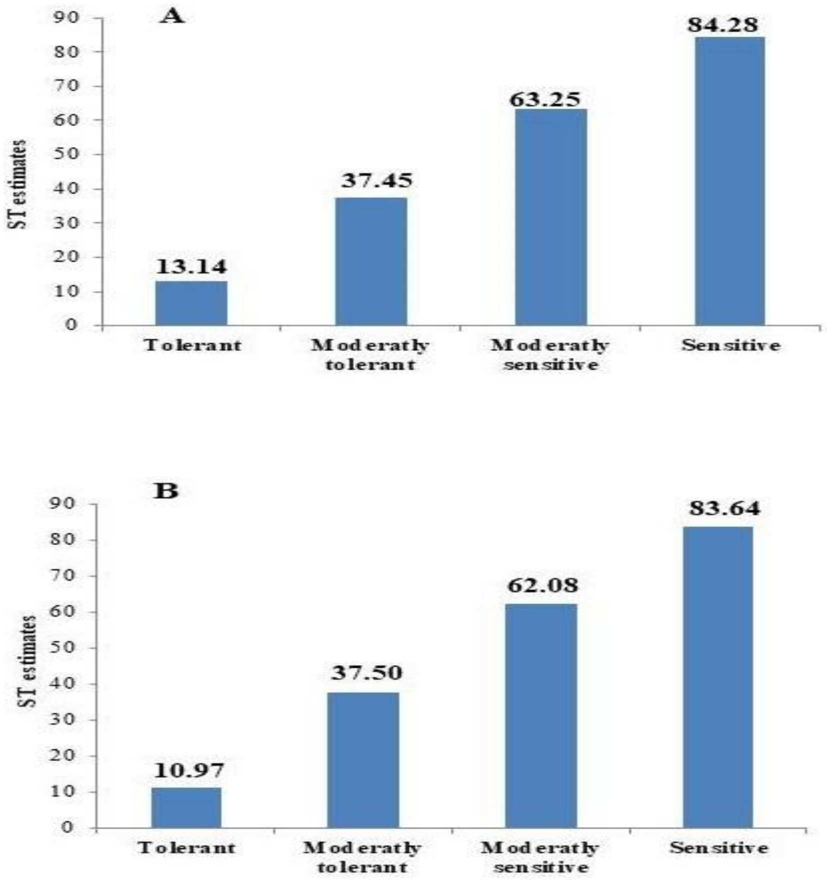

Figure S5. (A) Illustrated the representation of F_3 lines of Bobur*Altay 2000 based on the ST rankings.ST status of all the 274 genotypes. **(B)** Illustrated the representation of F_3 lines of Bobur*UZ-11CWA08 based on the ST rankings.ST status of all the 277 genotypes.

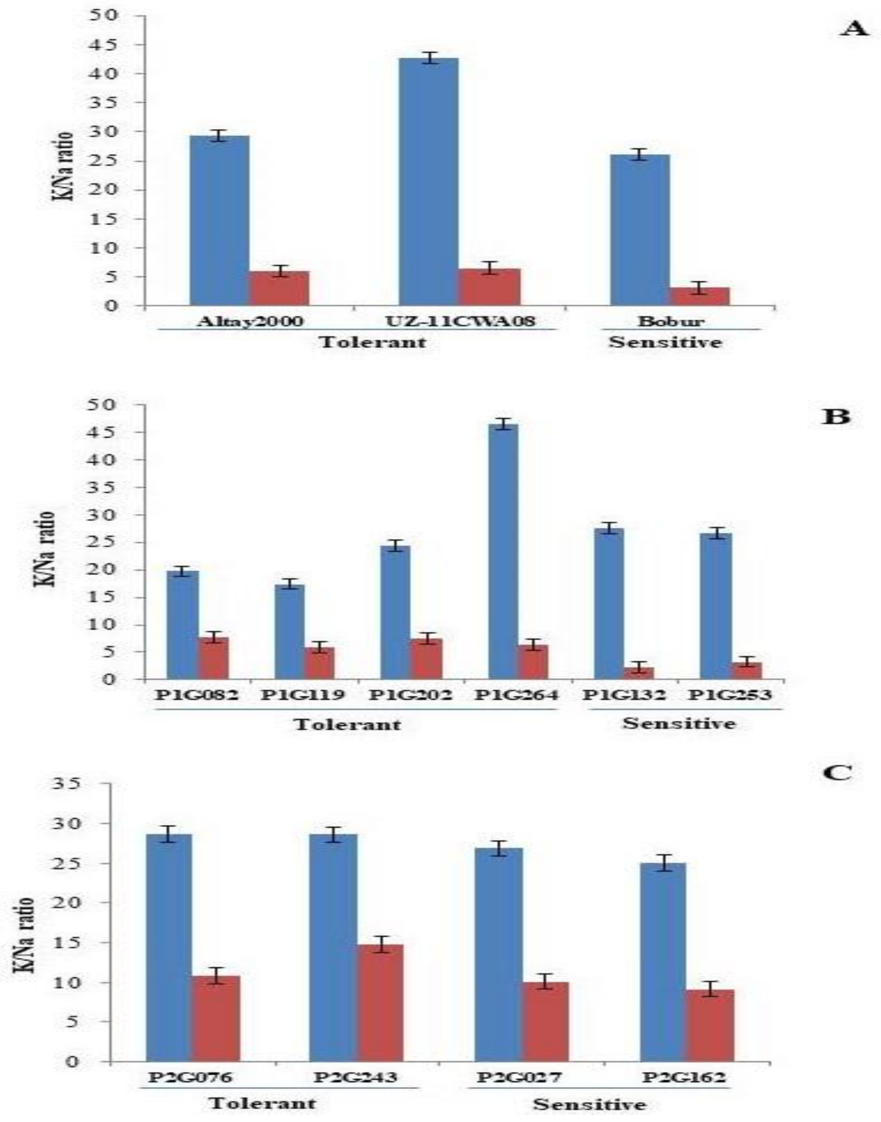

Figure S6. Effect of salt stress on Na^+/K^+ ratio. Parents **(A)**. Contrasting F_3 lines of cross Bobur*Altay2000 **(B)** and of cross Bobur* UZ-11CWA08 **(C)**. non-saline (blue) and saline (red) conditions.

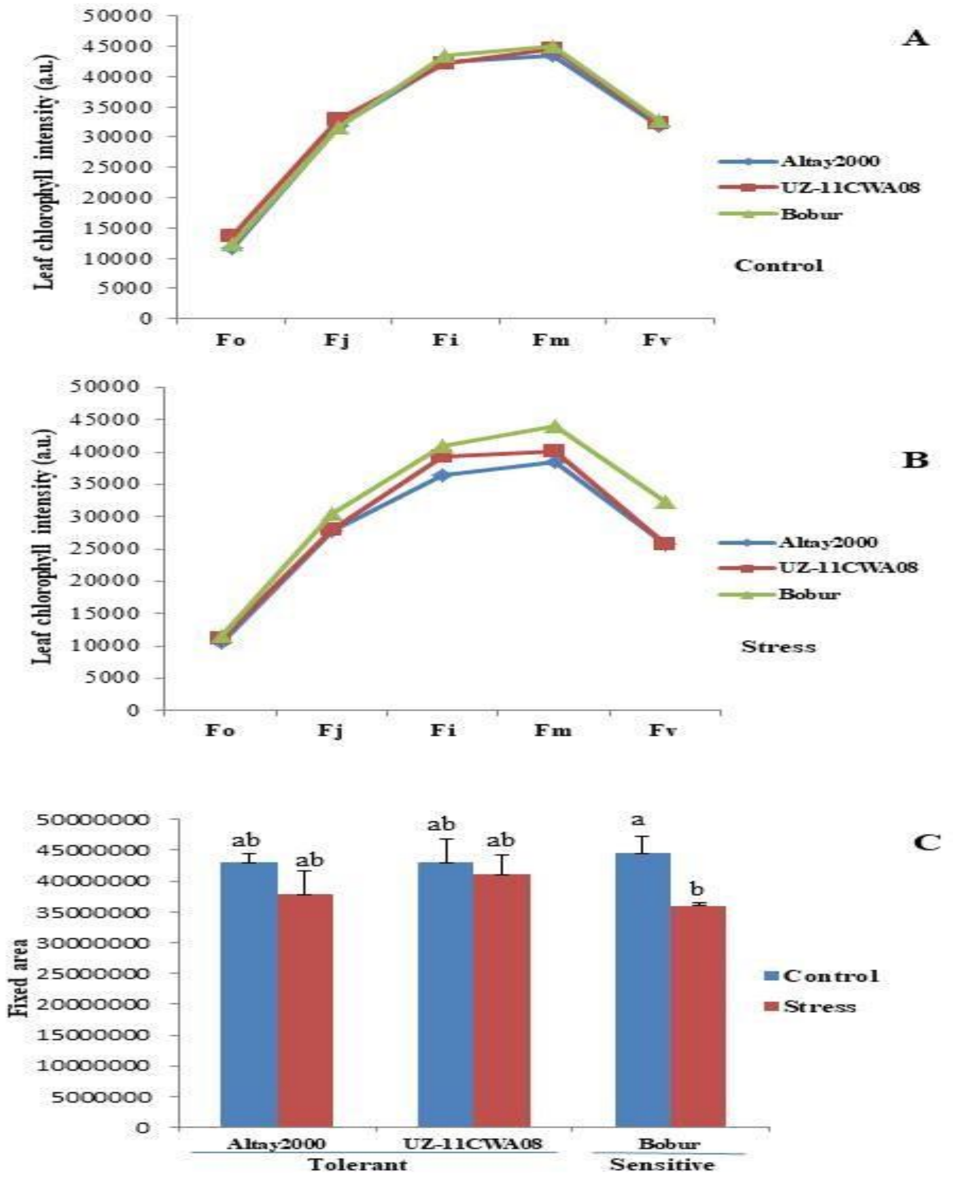

Figure S7. Effect of salt stress on the chlorophyll a fluorescence and OJIP test parameters of light-adapted leaves of two salt-tolerant (Altay2000, UZ-11CWA-8) and salt-sensitive wheat genotype (Bobur) identified in this study. **(A)** and **(B)** Chlorophyll a fluorescence kinetics curve under control and stress conditions respectively (Fo = fluorescence intensity at 50 ls; Fj = fluorescence intensity at J-step (at 2 ms); Fi = fluorescence intensity at i-step (at 60 ms);Fm = maximal fluorescence intensity; Fv = maximal variable fluorescence). **(C)** Fix area representing the area above the chlorophyll fluorescence curve between Fo and Fm (size of the plastoquinone pool). Letters on the error bars indicate comparison of the genotype means under control and salt stress conditions. Means with the same letter are not significantly different from each other. Error bars represent SEs. Different letters denote statistically significant differences among the genotypes at $P<0.05$ level detected by Duncan multiple range test.

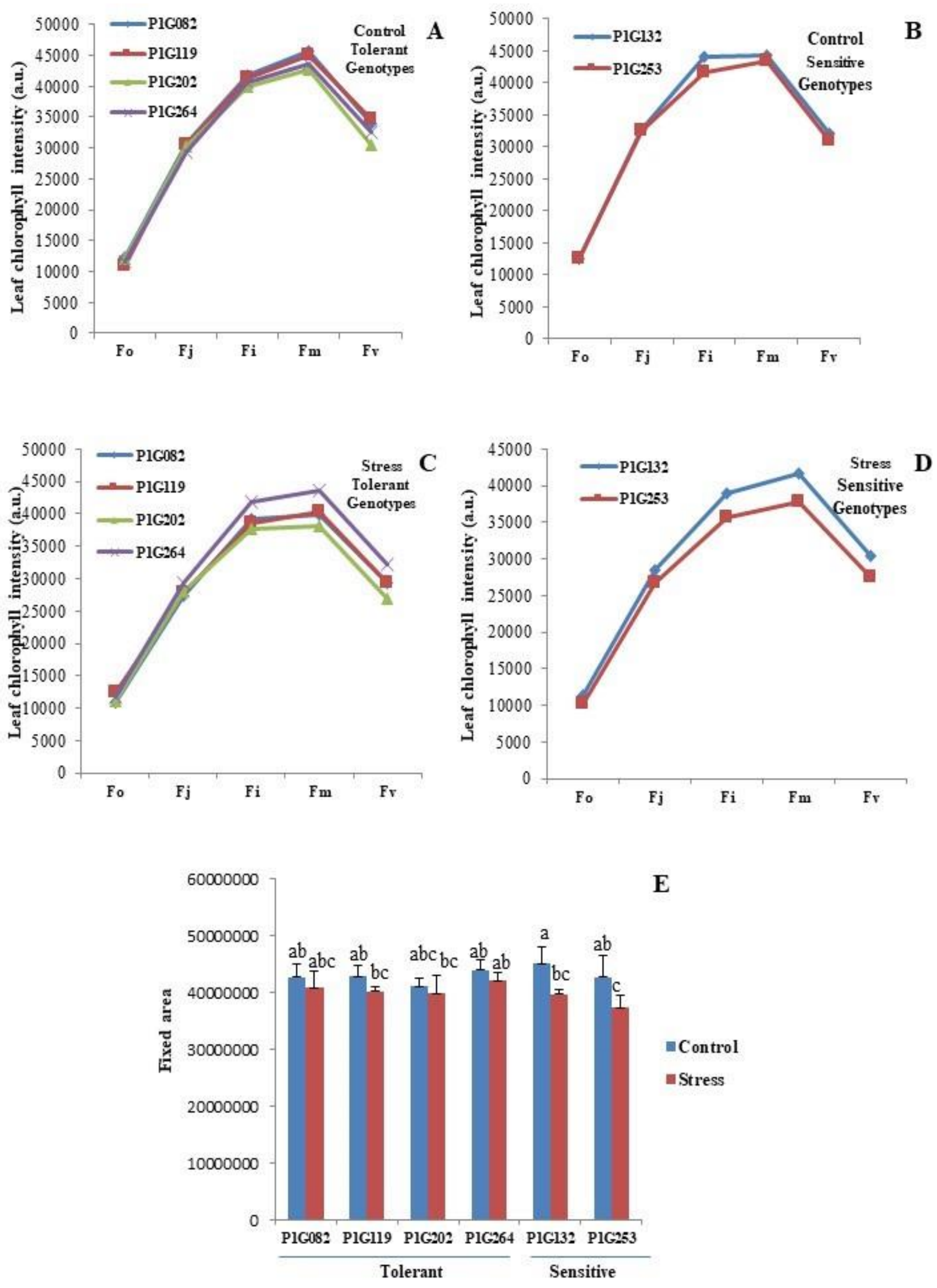

Figure S8. Effect of salt stress on the chlorophyll a fluorescence and OJIP test parameters of light-adapted leaves of contrasting F_3 lines of cross Bobur*Altay2000 identified in this study. **(A)** Salt-tolerant genotypes under control conditions. **(B)** Salt-sensitive genotypes under control conditions, **(C)** Salt- tolerant genotypes under stress conditions . **(D)** Salt-sensitive genotypes under stress conditions Chlorophyll a fluorescence kinetics curve(Fo = fluorescence intensity at 50 ls; Fj = fluorescence intensity at J-step (at 2 ms); Fi = fluorescence intensity at i-step (at 60 ms);Fm = maximal fluorescence intensity;Fv = maximal variable fluorescence). **(E)** Fix area representing the area above the chlorophyll fluorescence curve between Fo and Fm (size of the plastoquinone pool). Letters on the error bars indicate comparison of the genotype means under control and salt stress conditions. Means with the same letter are not significantly different from each other. Error bars represent SEs. Different letters denote statistically significant differences among the genotypes at P<0.05 level detected by Duncan multiple range test.

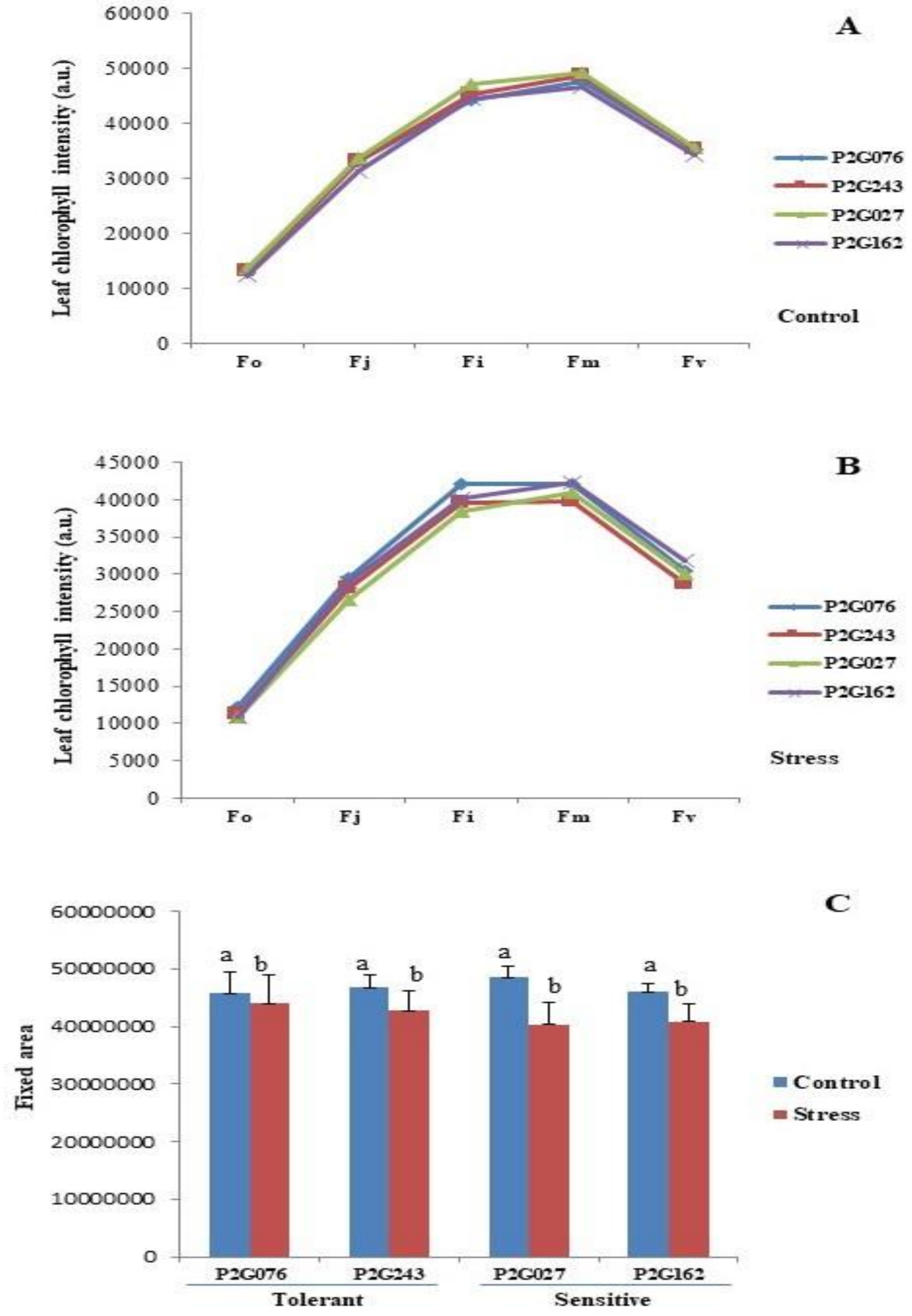

Figure S9. Effect of salt stress on the chlorophyll a fluorescence and OJIP test parameters of light-adapted leaves of contrasting F_3 lines of cross Bobur*UZ-11CWA08. identified in this study. **(A)** and **(B)** Chlorophyll a fluorescence kinetics curve under control and stress conditions respectively (Fo = fluorescence intensity at 50 ls; Fj = fluorescence intensity at J-step (at 2 ms); Fi = fluorescence intensity at i-step (at 60 ms);Fm = maximal fluorescence intensity; Fv = maximal variable fluorescence). **(C)** Fix area representing the area above the chlorophyll fluorescence curve between Fo and Fm (size of the plastoquinone pool). Letters on the error bars indicate comparison of the genotype means under control and salt stress conditions. Means with the same letter are not significantly different from each other. Error bars represent SEs. Different letters denote statistically significant differences among the genotypes at P<0.05 level detected by Duncan multiple range test.

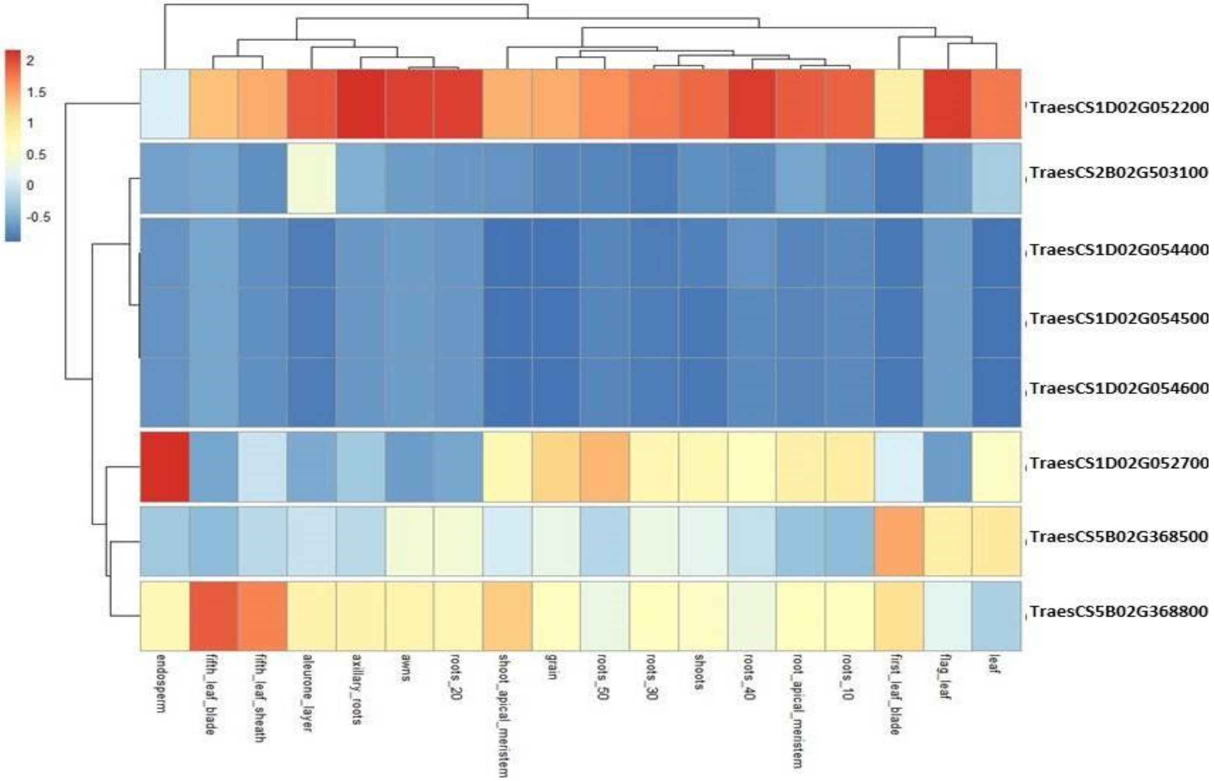

Figure S10. Expression patterns of selected candidate genes for salinity tolerance within different tissues of wheat. Expression data were obtained from the Wheat Gmap database (https://www.wheatgmap.org/expression/search/gene/) and are presented as a heatmap of transcripts per kilobase million (TPM) values.

Table S1. F-values and significance levels of the combined ANOVA. The seedling stage traits in the hydroponic tests are analysed for both populations together.

**Source of Variance**	**DF**	**SFW (F-Value)**	**SDW (F-Value)**	**RFW (F-Value)**	**RDW (F-Value)**
**Salt-Treatment (ST)**	1	3181.97 ***	1887.43 ***	1760.77 ***	1350.48 ***
**Population (P)**	1	118.41 ***	140.15 ***	57.81 ***	128.08 ***
**ST*P**	1	136.96 ***	12.70 ***	64.04 ***	74.13 ***
**Genotypes within Population (G(P))**	549	1.38 ***	1.83 ***	2.35 ***	3.09 ***
**ST*G(P)**	543	3.10 ***	1.16 *	0.81 ns	0.48 ns
		MS	MS	MS	MS
**Error**	1648	1.235858	0.033956	0.674057	0.016304

Note: F-values are shown; significance levels p: * $p \leq 0.05$; *** $p \leq 0.001$; ns not significant; DF degree of freedom; SFW shoot fresh weight; SDW shoot dry weight; RFW root fresh weight; RDW root dry weight.

Table S2. ANOVA and descriptive statistics on phenotypic traits of parents evaluated in two water salinity treatment (T).

	Altay2000								UZ-11CWA08								Bobur							
	SFW		SDW		RFW		RDW		SFW		SDW		RFW		RDW		SFW		SDW		RFW		RDW	
	C	S	C	S	C	S	C	S	C	S	C	S	C	S	C	S	C	S	C	S	C	S	C	S
**SD**	0.8	0.5	0.1	0.1	0.3	0.2	0.09	0.08	0.8	0.7	0.07	0.06	0.4	0.4	0.02	0.05	0.03	0.4	0.08	0.095	0.4	0.2	0.05	0.04
**Mean**	3.7	3.2	1.7	1.6	2.2	2.3	1.4	1.5	7.1	2.9	2.05	1.5	3.3	2.3	1.5	1.3	7.6	2.7	2.2	1.5	2.6	2.1	1.4	1.4
**Min**	2.8	2.8	1.6	1.5	1.9	2.1	1.4	1.4	6.3	2.1	1.9	1.4	2.9	1.7	1.5	1.3	7.5	2.3	2.1	1.4	2.1	1.9	1.4	1.4
**Max**	4.5	3.9	1.9	1.7	2.6	2.6	1.5	1.5	7.9	3.5	2.1	1.6	3.8	2.7	1.5	1.4	7.6	3.2	2.3	1.6	3.08	2.4	1.5	1.4
**Range**	1.6	1.07	0.3	0.2	0.6	0.5	0.1	0.1	1.6	1.4	0.1	0.1	0.8	0.9	0.04	0.1	0.07	0.9	0.1	0.1	0.8	0.5	0.1	0.08
**Variance**	0.7	0.3	0.02	0.01	0.1	0.07	0.009	0.006	0.6	0.5	0.005	0.004	0.1	0.2	0.0004	0.002	0.001	0.2	0.007	0.009	0.1	0.08	0.002	0.001
**CV%**	22.3	17.1	9.03	6.7	15.8	11.9	6.6	5.3	11.4	24.4	3.6	4.2	13.01	20.6	1.2	3.9	0.4	17.7	3.8	6.1	16.8	13.7	3.3	2.7

Note:- C, Control; S Stress; SD Standard deviation; CV% Coefficient of Variation.

Table S3. ANOVA and descriptive statistics on phenotypic traits of parents evaluated in two water salinity treatment (T).

	F_3 lines of cross Bobur*Altay2000								F_3 lines of cross Bobur* UZ-11CWA08							
	SFW		SDW		RFW		RDW		SFW		SDW		RFW		RDW	
	C	S	C	S	C	S	C	S	C	S	C	S	C	S	C	S
**SD**	2.3	0.8	0.2	0.1	0.8	0.55	0.11	0.09	1.8	0.6	0.2	0.1	1.3	0.5	0.1	0.1
**Mean**	8.4	3.2	2.02	1.6	3.5	2.50	1.56	1.4	6.5	3.1	1.8	1.5	4.06	2.6	1.5	1.3
**Min**	1.5	1.4	1.3	1.2	1.2	1.37	1.17	1	2.2	1.8	1.2	1.1	1.7	1.05	1.0	1.04
**Max**	17.2	6.6	3.3	2.1	8.01	5.38	2.49	2.3	13.5	5.8	3.1	2.3	10.9	5.7	2.4	1.88
**Range**	15.6	5.1	2.07	0.9	6.7	4.01	1.32	1.3	11.3	4	1.9	1.1	9.2	4.6	1.3	0.84
**Variance**	5.7	0.7	0.07	0.02	0.7	0.30	0.01	0.008	3.4	0.4	0.05	0.02	1.7	0.3	0.03	0.01
**CV%**	28.3	27.07	13.1	9.6	24.7	22.16	7.34	6.2	28.3	20.5	12.2	9.2	32.4	22.4	12.1	9.2
**h^2% BS**	**51.90**		**40.84**		**27.54**		**26.08**		**34.50**		**26.30**		**19.02**		**12.32**	

Table S4. Overall ST Ranking for 274 F_3 lines of cross Bobur*Altay2000.

Genoname	Overall ST Ranking	Status
P1G200	0.625651721	Tolerant
P1G094	1.772679875	Tolerant
P1G204	3.910323253	Tolerant
P1G104	3.962460897	Tolerant
P1G153	4.483837331	Tolerant
P1G032	4.640250261	Tolerant
P1G229	4.640250261	Tolerant
P1G036	5.735140772	Tolerant
P1G097	5.735140772	Tolerant
P1G147	5.891553702	Tolerant
P1G135	7.24713243	Tolerant
P1G264	7.40354536	Tolerant
P1G107	8.02919708	Tolerant
P1G171	8.02919708	Tolerant
P1G119	8.081334724	Tolerant
P1G029	9.541188738	Tolerant
P1G207	10.47966632	Tolerant
P1G015	10.58394161	Tolerant
P1G201	11.57455683	Tolerant
P1G219	12.20020855	Tolerant
P1G082	12.30448384	Tolerant
P1G166	13.19082377	Tolerant
P1G202	13.39937435	Tolerant
P1G243	13.55578728	Tolerant
P1G185	14.07716371	Tolerant
P1G006	14.33785193	Tolerant
P1G028	14.75495308	Tolerant
P1G074	14.91136601	Tolerant
P1G175	15.06777894	Tolerant
P1G043	16.21480709	Tolerant
P1G144	16.31908238	Tolerant
P1G105	17.15328467	Tolerant
P1G246	17.72679875	Tolerant
P1G123	17.93534932	Tolerant
P1G058	18.5088634	Tolerant
P1G247	18.66527633	Tolerant
P1G127	19.60375391	Tolerant
P1G112	20.4379562	Tolerant

P1G059	20.49009385	Tolerant
P1G111	20.54223149	Tolerant
P1G035	20.90719499	Tolerant
P1G026	20.95933264	Tolerant
P1G046	20.95933264	Tolerant
P1G022	21.79353493	Tolerant
P1G258	22.00208551	Tolerant
P1G072	22.94056309	Tolerant
P1G005	23.25338895	Tolerant
P1G268	23.35766423	Tolerant
P1G262	23.72262774	Tolerant
P1G158	23.77476538	Tolerant
P1G093	24.29614181	Tolerant
P1G136	24.4004171	Tolerant
P1G221	25.75599583	Moderate Tolerant
P1G096	26.22523462	Moderate Tolerant
P1G084	26.48592284	Moderate Tolerant
P1G075	26.64233577	Moderate Tolerant
P1G257	26.85088634	Moderate Tolerant
P1G041	26.95516163	Moderate Tolerant
P1G238	27.68508863	Moderate Tolerant
P1G232	27.78936392	Moderate Tolerant
P1G162	27.99791449	Moderate Tolerant
P1G099	28.20646507	Moderate Tolerant
P1G106	28.46715328	Moderate Tolerant
P1G033	28.51929093	Moderate Tolerant
P1G216	28.57142857	Moderate Tolerant
P1G208	28.88425443	Moderate Tolerant
P1G047	29.04066736	Moderate Tolerant
P1G169	29.19708029	Moderate Tolerant
P1G228	29.5620438	Moderate Tolerant
P1G235	29.66631908	Moderate Tolerant
P1G065	29.82273201	Moderate Tolerant
P1G086	29.87486966	Moderate Tolerant
P1G071	29.9270073	Moderate Tolerant
P1G140	30.03128259	Moderate Tolerant
P1G225	30.13555787	Moderate Tolerant
P1G049	31.07403545	Moderate Tolerant
P1G011	31.43899896	Moderate Tolerant
P1G056	31.64754953	Moderate Tolerant
P1G100	32.06465068	Moderate Tolerant

P1G020	32.32533889	Moderate Tolerant
P1G227	32.48175182	Moderate Tolerant
P1G007	32.84671533	Moderate Tolerant
P1G030	32.89885297	Moderate Tolerant
P1G087	34.20229406	Moderate Tolerant
P1G250	34.2544317	Moderate Tolerant
P1G260	34.93222106	Moderate Tolerant
P1G008	35.03649635	Moderate Tolerant
P1G259	35.14077164	Moderate Tolerant
P1G237	35.29718457	Moderate Tolerant
P1G108	35.66214807	Moderate Tolerant
P1G271	35.71428571	Moderate Tolerant
P1G146	35.87069864	Moderate Tolerant
P1G092	36.80917623	Moderate Tolerant
P1G134	36.96558916	Moderate Tolerant
P1G197	37.64337852	Moderate Tolerant
P1G052	37.90406674	Moderate Tolerant
P1G060	38.11261731	Moderate Tolerant
P1G180	38.16475495	Moderate Tolerant
P1G165	38.32116788	Moderate Tolerant
P1G244	38.42544317	Moderate Tolerant
P1G174	38.47758081	Moderate Tolerant
P1G002	38.89468196	Moderate Tolerant
P1G025	39.10323253	Moderate Tolerant
P1G016	39.67674661	Moderate Tolerant
P1G051	39.67674661	Moderate Tolerant
P1G193	39.7810219	Moderate Tolerant
P1G113	39.83315954	Moderate Tolerant
P1G068	40.04171011	Moderate Tolerant
P1G274	40.56308655	Moderate Tolerant
P1G018	40.87591241	Moderate Tolerant
P1G031	41.13660063	Moderate Tolerant
P1G242	41.29301356	Moderate Tolerant
P1G209	41.86652763	Moderate Tolerant
P1G076	43.22210636	Moderate Tolerant
P1G152	43.274244	Moderate Tolerant
P1G013	43.69134515	Moderate Tolerant
P1G191	44.00417101	Moderate Tolerant
P1G222	44.42127216	Moderate Tolerant
P1G233	44.42127216	Moderate Tolerant
P1G004	44.62982273	Moderate Tolerant

P1G142	44.89051095	Moderate Tolerant
P1G048	45.09906152	Moderate Tolerant
P1G021	45.6725756	Moderate Tolerant
P1G045	46.0375391	Moderate Tolerant
P1G249	46.24608968	Moderate Tolerant
P1G138	46.76746611	Moderate Tolerant
P1G083	47.08029197	Moderate Tolerant
P1G101	47.08029197	Moderate Tolerant
P1G137	47.49739312	Moderate Tolerant
P1G252	48.01876955	Moderate Tolerant
P1G241	48.12304484	Moderate Tolerant
P1G217	48.17518248	Moderate Tolerant
P1G023	48.38373306	Moderate Tolerant
P1G196	48.38373306	Moderate Tolerant
P1G251	48.54014599	Moderate Tolerant
P1G218	48.64442127	Moderate Tolerant
P1G256	48.74869656	Moderate Sensitive
P1G110	48.90510949	Moderate Sensitive
P1G179	49.37434828	Moderate Sensitive
P1G089	50.3649635	Moderate Sensitive
P1G114	50.57351408	Moderate Sensitive
P1G194	50.72992701	Moderate Sensitive
P1G181	50.83420229	Moderate Sensitive
P1G265	51.1991658	Moderate Sensitive
P1G198	51.45985401	Moderate Sensitive
P1G205	51.87695516	Moderate Sensitive
P1G150	52.34619395	Moderate Sensitive
P1G017	52.7632951	Moderate Sensitive
P1G103	53.02398332	Moderate Sensitive
P1G010	53.44108446	Moderate Sensitive
P1G155	53.49322211	Moderate Sensitive
P1G090	53.70177268	Moderate Sensitive
P1G230	54.64025026	Moderate Sensitive
P1G063	54.79666319	Moderate Sensitive
P1G211	55.05735141	Moderate Sensitive
P1G143	55.16162669	Moderate Sensitive
P1G080	55.73514077	Moderate Sensitive
P1G145	55.78727842	Moderate Sensitive
P1G210	56.77789364	Moderate Sensitive
P1G183	57.29927007	Moderate Sensitive
P1G053	57.455683	Moderate Sensitive

P1G057	57.71637122	Moderate Sensitive
P1G245	57.97705944	Moderate Sensitive
P1G117	58.34202294	Moderate Sensitive
P1G102	58.70698644	Moderate Sensitive
P1G040	59.22836288	Moderate Sensitive
P1G184	59.22836288	Moderate Sensitive
P1G009	59.54118874	Moderate Sensitive
P1G234	59.80187696	Moderate Sensitive
P1G220	60.06256517	Moderate Sensitive
P1G273	60.11470282	Moderate Sensitive
P1G019	60.47966632	Moderate Sensitive
P1G070	60.58394161	Moderate Sensitive
P1G037	61.00104275	Moderate Sensitive
P1G067	61.57455683	Moderate Sensitive
P1G003	62.25234619	Moderate Sensitive
P1G128	62.51303441	Moderate Sensitive
P1G131	62.87799791	Moderate Sensitive
P1G064	62.93013556	Moderate Sensitive
P1G187	64.02502607	Moderate Sensitive
P1G272	64.33785193	Moderate Sensitive
P1G190	64.44212722	Moderate Sensitive
P1G027	65.53701773	Moderate Sensitive
P1G141	66.00625652	Moderate Sensitive
P1G055	66.73618352	Moderate Sensitive
P1G085	66.78832117	Moderate Sensitive
P1G125	66.78832117	Moderate Sensitive
P1G115	66.84045881	Moderate Sensitive
P1G039	67.10114703	Moderate Sensitive
P1G157	67.20542231	Moderate Sensitive
P1G223	67.36183525	Moderate Sensitive
P1G177	67.83107404	Moderate Sensitive
P1G189	68.03962461	Moderate Sensitive
P1G118	68.1438999	Moderate Sensitive
P1G195	68.92596455	Moderate Sensitive
P1G038	68.97810219	Moderate Sensitive
P1G124	69.39520334	Moderate Sensitive
P1G120	69.44734098	Moderate Sensitive
P1G062	69.49947862	Moderate Sensitive
P1G160	69.76016684	Moderate Sensitive
P1G206	69.81230448	Moderate Sensitive
P1G042	70.12513034	Moderate Sensitive

P1G248	71.11574557	Moderate Sensitive
P1G173	71.79353493	Moderate Sensitive
P1G239	71.84567258	Moderate Sensitive
P1G214	71.89781022	Moderate Sensitive
P1G024	72.21063608	Moderate Sensitive
P1G156	72.8362878	Moderate Sensitive
P1G203	72.8362878	Moderate Sensitive
P1G267	72.94056309	Moderate Sensitive
P1G088	73.04483837	Moderate Sensitive
P1G215	73.14911366	Moderate Sensitive
P1G176	73.30552659	Moderate Sensitive
P1G236	73.61835245	Moderate Sensitive
P1G255	73.77476538	Moderate Sensitive
P1G186	74.24400417	Moderate Sensitive
P1G054	74.55683003	Moderate Sensitive
P1G133	74.97393118	Moderate Sensitive
P1G213	74.97393118	Moderate Sensitive
P1G095	75.28675704	Sensitive
P1G240	75.49530761	Sensitive
P1G269	75.91240876	Sensitive
P1G012	76.01668405	Sensitive
P1G079	76.06882169	Sensitive
P1G122	76.64233577	Sensitive
P1G254	77.00729927	Sensitive
P1G044	77.05943691	Sensitive
P1G149	77.21584984	Sensitive
P1G266	77.68508863	Sensitive
P1G001	78.362878	Sensitive
P1G168	78.51929093	Sensitive
P1G161	78.93639208	Sensitive
P1G014	79.09280501	Sensitive
P1G159	79.40563087	Sensitive
P1G154	79.87486966	Sensitive
P1G073	80.34410845	Sensitive
P1G091	80.34410845	Sensitive
P1G081	80.55265902	Sensitive
P1G121	80.96976017	Sensitive
P1G199	82.11678832	Sensitive
P1G263	82.37747654	Sensitive
P1G151	82.42961418	Sensitive
P1G170	82.74244004	Sensitive

P1G270	83.10740355	Sensitive
P1G261	83.68091762	Sensitive
P1G098	83.8894682	Sensitive
P1G126	83.8894682	Sensitive
P1G109	83.94160584	Sensitive
P1G116	85.29718457	Sensitive
P1G167	86.1835245	Sensitive
P1G164	86.33993743	Sensitive
P1G077	86.44421272	Sensitive
P1G148	86.44421272	Sensitive
P1G231	87.06986444	Sensitive
P1G129	87.33055266	Sensitive
P1G212	87.79979145	Sensitive
P1G178	88.32116788	Sensitive
P1G050	89.83315954	Sensitive
P1G078	90.71949948	Sensitive
P1G069	90.9801877	Sensitive
P1G172	91.3451512	Sensitive
P1G182	91.39728884	Sensitive
P1G163	91.91866528	Sensitive
P1G034	92.544317	Sensitive
P1G253	93.16996872	Sensitive
P1G192	94.31699687	Sensitive
P1G130	94.83837331	Sensitive
P1G132	95.25547445	Sensitive
P1G061	95.77685089	Sensitive
P1G188	95.98540146	Sensitive

Table S5. Overall ST Ranking for 274 F_3 lines of cross Bobur* UZ-11CWA08.

Genoname	Overall ST Ranking	Status
P2G143	3.713254255	Tolerant
P2G223	4.074265085	Tolerant
P2G142	4.332129964	Tolerant
P2G067	6.291903043	Tolerant
P2G087	6.291903043	Tolerant
P2G047	7.013924703	Tolerant
P2G178	8.406395049	Tolerant
P2G181	8.561113976	Tolerant
P2G227	8.664259928	Tolerant
P2G164	9.437854564	Tolerant
P2G003	9.541000516	Tolerant
P2G230	10.62403301	Tolerant
P2G220	10.72717896	Tolerant
P2G207	10.93347086	Tolerant
P2G149	11.60391955	Tolerant
P2G076	11.65549252	Tolerant
P2G216	12.53223311	Tolerant
P2G221	12.53223311	Tolerant
P2G053	12.63537906	Tolerant
P2G214	13.04796287	Tolerant
P2G243	13.25425477	Tolerant
P2G135	13.51211965	Tolerant
P2G145	13.66683858	Tolerant
P2G179	13.66683858	Tolerant
P2G113	13.76998453	Tolerant
P2G132	14.18256833	Tolerant
P2G154	14.44043321	Tolerant
P2G235	14.49200619	Tolerant
P2G273	14.95616297	Tolerant
P2G081	15.26560083	Tolerant
P2G174	15.57503868	Tolerant
P2G219	16.50335224	Tolerant
P2G199	16.86436307	Tolerant
P2G251	17.019082	Tolerant
P2G184	17.2253739	Tolerant
P2G232	17.58638473	Tolerant
P2G190	17.68953069	Tolerant
P2G276	19.59773079	Tolerant

P2G168	20.26817947	Tolerant
P2G072	20.68076328	Tolerant
P2G055	21.19649304	Tolerant
P2G177	21.29963899	Tolerant
P2G118	21.45435792	Tolerant
P2G204	21.7122228	Tolerant
P2G044	22.3310985	Tolerant
P2G264	22.53739041	Tolerant
P2G234	22.64053636	Tolerant
P2G261	22.74368231	Tolerant
P2G176	23.25941207	Tolerant
P2G277	25.27075812	Moderate Tolerant
P2G086	25.42547705	Moderate Tolerant
P2G171	25.7349149	Moderate Tolerant
P2G239	25.99277978	Moderate Tolerant
P2G159	26.09592573	Moderate Tolerant
P2G007	26.92109335	Moderate Tolerant
P2G213	27.48839608	Moderate Tolerant
P2G228	27.48839608	Moderate Tolerant
P2G147	27.69468798	Moderate Tolerant
P2G202	27.74626096	Moderate Tolerant
P2G203	27.84940691	Moderate Tolerant
P2G140	27.90097989	Moderate Tolerant
P2G017	28.00412584	Moderate Tolerant
P2G186	28.41670964	Moderate Tolerant
P2G187	28.5198556	Moderate Tolerant
P2G272	29.24187726	Moderate Tolerant
P2G161	29.39659618	Moderate Tolerant
P2G215	29.86075297	Moderate Tolerant
P2G253	30.11861784	Moderate Tolerant
P2G194	30.17019082	Moderate Tolerant
P2G116	30.27333677	Moderate Tolerant
P2G250	30.37648272	Moderate Tolerant
P2G153	31.09850438	Moderate Tolerant
P2G192	31.15007736	Moderate Tolerant
P2G052	31.30479629	Moderate Tolerant
P2G120	32.07839092	Moderate Tolerant
P2G151	32.1299639	Moderate Tolerant
P2G150	32.74883961	Moderate Tolerant
P2G013	33.00670449	Moderate Tolerant
P2G131	33.67715317	Moderate Tolerant

P2G083	33.78029912	Moderate Tolerant
P2G008	33.88344507	Moderate Tolerant
P2G237	34.038164	Moderate Tolerant
P2G191	34.14130995	Moderate Tolerant
P2G229	34.29602888	Moderate Tolerant
P2G146	34.65703971	Moderate Tolerant
P2G160	34.65703971	Moderate Tolerant
P2G048	34.86333161	Moderate Tolerant
P2G155	34.96647757	Moderate Tolerant
P2G172	35.22434244	Moderate Tolerant
P2G246	35.5337803	Moderate Tolerant
P2G212	35.58535327	Moderate Tolerant
P2G256	35.63692625	Moderate Tolerant
P2G091	35.84321815	Moderate Tolerant
P2G241	35.84321815	Moderate Tolerant
P2G254	35.99793708	Moderate Tolerant
P2G252	36.82310469	Moderate Tolerant
P2G193	37.0293966	Moderate Tolerant
P2G097	37.08096957	Moderate Tolerant
P2G170	37.18411552	Moderate Tolerant
P2G245	37.49355338	Moderate Tolerant
P2G249	37.95771016	Moderate Tolerant
P2G205	38.11242909	Moderate Tolerant
P2G031	38.62815884	Moderate Tolerant
P2G244	38.62815884	Moderate Tolerant
P2G079	38.67973182	Moderate Tolerant
P2G152	38.78287777	Moderate Tolerant
P2G240	38.78287777	Moderate Tolerant
P2G196	38.88602372	Moderate Tolerant
P2G260	39.35018051	Moderate Tolerant
P2G026	40.07220217	Moderate Tolerant
P2G034	40.07220217	Moderate Tolerant
P2G035	40.17534812	Moderate Tolerant
P2G236	40.17534812	Moderate Tolerant
P2G271	40.22692109	Moderate Tolerant
P2G165	40.48478597	Moderate Tolerant
P2G175	40.58793192	Moderate Tolerant
P2G210	40.94894275	Moderate Tolerant
P2G033	41.20680763	Moderate Tolerant
P2G099	41.25838061	Moderate Tolerant
P2G050	41.36152656	Moderate Tolerant

P2G268	41.87725632	Moderate Tolerant
P2G274	42.03197524	Moderate Tolerant
P2G200	42.1351212	Moderate Tolerant
P2G225	43.16658071	Moderate Tolerant
P2G197	43.26972666	Moderate Tolerant
P2G270	43.52759154	Moderate Tolerant
P2G043	43.73388345	Moderate Tolerant
P2G242	44.14646725	Moderate Tolerant
P2G144	44.2496132	Moderate Tolerant
P2G163	44.55905106	Moderate Tolerant
P2G209	44.76534296	Moderate Tolerant
P2G266	44.97163486	Moderate Tolerant
P2G217	45.22949974	Moderate Tolerant
P2G158	46.36410521	Moderate Sensitive
P2G195	46.36410521	Moderate Sensitive
P2G054	46.46725116	Moderate Sensitive
P2G248	46.93140794	Moderate Sensitive
P2G169	46.98298092	Moderate Sensitive
P2G046	47.6534296	Moderate Sensitive
P2G032	47.70500258	Moderate Sensitive
P2G018	47.80814853	Moderate Sensitive
P2G231	48.01444043	Moderate Sensitive
P2G111	48.27230531	Moderate Sensitive
P2G201	49.09747292	Moderate Sensitive
P2G224	49.25219185	Moderate Sensitive
P2G014	49.3553378	Moderate Sensitive
P2G233	49.3553378	Moderate Sensitive
P2G188	49.66477566	Moderate Sensitive
P2G010	49.87106756	Moderate Sensitive
P2G157	50.69623517	Moderate Sensitive
P2G166	50.79938112	Moderate Sensitive
P2G129	51.00567303	Moderate Sensitive
P2G019	51.10881898	Moderate Sensitive
P2G069	51.52140278	Moderate Sensitive
P2G141	51.67612171	Moderate Sensitive
P2G128	52.14027849	Moderate Sensitive
P2G100	52.29499742	Moderate Sensitive
P2G066	52.60443528	Moderate Sensitive
P2G183	53.12016503	Moderate Sensitive
P2G038	53.27488396	Moderate Sensitive
P2G077	53.48117586	Moderate Sensitive

P2G030	54.4610624	Moderate Sensitive
P2G064	54.66735431	Moderate Sensitive
P2G036	54.92521919	Moderate Sensitive
P2G122	55.02836514	Moderate Sensitive
P2G080	55.44094894	Moderate Sensitive
P2G112	55.80195977	Moderate Sensitive
P2G049	56.00825168	Moderate Sensitive
P2G148	56.1629706	Moderate Sensitive
P2G117	56.42083548	Moderate Sensitive
P2G108	56.62712739	Moderate Sensitive
P2G016	57.76173285	Moderate Sensitive
P2G269	58.01959773	Moderate Sensitive
P2G029	58.84476534	Moderate Sensitive
P2G063	59.3604951	Moderate Sensitive
P2G173	59.51521403	Moderate Sensitive
P2G265	60.59824652	Moderate Sensitive
P2G068	60.64981949	Moderate Sensitive
P2G023	61.78442496	Moderate Sensitive
P2G057	62.45487365	Moderate Sensitive
P2G011	62.60959257	Moderate Sensitive
P2G226	62.60959257	Moderate Sensitive
P2G059	62.71273853	Moderate Sensitive
P2G114	63.07374936	Moderate Sensitive
P2G024	63.12532233	Moderate Sensitive
P2G123	63.22846828	Moderate Sensitive
P2G258	63.22846828	Moderate Sensitive
P2G206	63.38318721	Moderate Sensitive
P2G062	63.58947911	Moderate Sensitive
P2G101	63.69262506	Moderate Sensitive
P2G134	63.89891697	Moderate Sensitive
P2G263	64.20835482	Moderate Sensitive
P2G167	64.62093863	Moderate Sensitive
P2G107	65.80711707	Moderate Sensitive
P2G002	66.27127385	Moderate Sensitive
P2G137	66.32284683	Moderate Sensitive
P2G257	66.42599278	Moderate Sensitive
P2G267	66.68385766	Moderate Sensitive
P2G056	66.73543063	Moderate Sensitive
P2G119	67.35430634	Moderate Sensitive
P2G136	67.50902527	Moderate Sensitive
P2G039	68.48891181	Moderate Sensitive

P2G106	68.59205776	Moderate Sensitive
P2G060	69.67509025	Moderate Sensitive
P2G262	69.67509025	Moderate Sensitive
P2G009	69.98452811	Moderate Sensitive
P2G078	70.08767406	Moderate Sensitive
P2G084	70.8612687	Moderate Sensitive
P2G124	72.09902011	Moderate Sensitive
P2G275	72.30531202	Moderate Sensitive
P2G185	72.82104177	Moderate Sensitive
P2G198	72.87261475	Moderate Sensitive
P2G074	73.1820526	Moderate Sensitive
P2G045	73.59463641	Moderate Sensitive
P2G211	73.64620939	Moderate Sensitive
P2G040	74.78081485	Moderate Sensitive
P2G025	74.83238783	Moderate Sensitive
P2G037	75.70912842	Sensitive
P2G180	75.81227437	Sensitive
P2G105	76.07013925	Sensitive
P2G189	76.32800413	Sensitive
P2G126	76.53429603	Sensitive
P2G222	76.79216091	Sensitive
P2G255	76.79216091	Sensitive
P2G006	77.61732852	Sensitive
P2G102	77.77204745	Sensitive
P2G156	77.97833935	Sensitive
P2G015	78.59721506	Sensitive
P2G104	78.80350696	Sensitive
P2G182	79.06137184	Sensitive
P2G238	79.16451779	Sensitive
P2G090	79.62867457	Sensitive
P2G051	79.93811243	Sensitive
P2G092	79.93811243	Sensitive
P2G012	80.09283136	Sensitive
P2G022	80.29912326	Sensitive
P2G138	80.35069624	Sensitive
P2G082	81.43372873	Sensitive
P2G094	82.980918	Sensitive
P2G058	83.29035585	Sensitive
P2G004	83.96080454	Sensitive
P2G098	85.19855596	Sensitive
P2G070	85.25012893	Sensitive

P2G065	85.50799381	Sensitive
P2G088	85.61113976	Sensitive
P2G127	85.92057762	Sensitive
P2G130	86.53945333	Sensitive
P2G162	86.84889118	Sensitive
P2G005	87.77720474	Sensitive
P2G089	88.49922641	Sensitive
P2G115	88.86023724	Sensitive
P2G001	88.96338319	Sensitive
P2G028	89.32439402	Sensitive
P2G027	90.25270758	Sensitive
P2G041	90.61371841	Sensitive
P2G139	90.71686436	Sensitive
P2G073	91.07787519	Sensitive
P2G095	91.54203198	Sensitive
P2G042	92.36719959	Sensitive
P2G125	92.67663744	Sensitive
P2G020	92.77978339	Sensitive
P2G096	92.83135637	Sensitive

Table S6. F-values and significance levels of the two-way ANOVA. The seedling stage traits in the hydroponic tests are analysed for contrasting genotypes of both segregating populations and parents.

	Parents						Contrasting lines of F_3 lines of cross Bobur*Altay2000						Contrasting lines of F_3 lines of cross Bobur*UZ-11CWA08					
**Source of Variance**	DF	Na^+ content	K^+ content	proline accumulation	Chl. Content	Fixed area	DF	Na^+ content	K^+ content	proline accumulation	Chl. Content	Fixed area	DF	Na^+ content	K^+ content	proline accumulation	Chl. Content	Fixed area
**Genotypes (G)**	**2**	**45.84*** **	**4.356***	**0.532** ^{ns}	**1.433** ^{ns}	**0.722** ^{ns}	**5**	**12.605*** **	**4.099** **	**8.811*** **	**0.578** ^{ns}	**1.497** ^{ns}	**3**	**0.562** ^{ns}	**12.543*** **	**172.6*** **	**4.244***	**1.568** ^{ns}
**Salt-Treatment (ST)**	**1**	**299.37*** **	**331.66*** **	**27.852*** **	**45.33*** **	**15.88** **	**1**	**324.704*** **	**132.712*** **	**62.889*** **	**11.202** **	**16.254*** **	**1**	**74.427*** **	**87.723*** **	**795.2*** **	**95.453*** **	**60.495*** **
**G*ST**	**2**	**21.06*** **	**7.144** **	**13.035*** **	**3.896***	**2.066** ^{ns}	**5**	**9.875*** **	**14.235*** **	**15.908*** **	**0.266** ^{ns}	**0.985** ^{ns}	**3**	**1.122** ^{ns}	**4.673***	**309.7*** **	**13.396*** **	**0.587** ^{ns}
		**MS**	**MS**	**MS**	**MS**	**MS**		**MS**	**MS**	**MS**	**MS**	**MS**		**MS**	**MS**	**MS**	**MS**	**MS**
**Error**	**12**	**0.38**	**8.8**	**8.01**	**0.00595**	**7.867e+12**	**24**	**0.69**	**46**	**13.1**	**0.0297**	**5.319e+12**	**16**	**0.395**	**14.8**	**22**	**0.00324**	**2.891e+12**

Note: F-values are shown; significance levels p: * $p \leq 0.05$; ** $p \leq 0.01$; *** $p \leq 0.001$; ns, not significant; d.f, degree of freedom.

Table S7. Effect of salt stress on some energy fluxes of contrasting wheat genotypes

Energy fluxes	Genotypes	Control	Stress	Effect of salt (%)
Fm/Fo	Altay2000	3.73	3.64	2.41
	UZ-11CWA-8	3.29	3.26	0.91
	Bobur	3.68	3.81	-3.53
	P1G082	3.72	3.66	1.61
	P1G119	4.11	3.26	20.68
	P1G202	3.51	3.46	1.42
	P1G264	3.74	3.71	0.80
	P1G132	3.55	3.65	-2.82
	P1G253	3.49	3.67	-5.16
	P2G076	3.71	3.46	6.74
	P2G243	3.69	3.56	3.52
	P2G027	3.57	3.77	-5.60
	P2G162	3.79	3.88	-2.37
Fv/Fo	Altay2000	2.73	2.45	10.26
	UZ-11CWA-8	2.37	2.32	2.11
	Bobur	2.68	2.81	-4.85
	P1G082	2.76	2.68	2.90
	P1G119	3.18	2.37	25.47
	P1G202	2.51	2.45	2.39
	P1G264	2.81	2.72	3.20
	P1G132	2.56	2.66	-3.91
	P1G253	2.49	2.67	-7.23
	P2G076	2.75	2.51	8.73
	P2G243	2.68	2.57	4.10
	P2G027	2.57	2.77	-7.78
	P2G162	2.79	2.91	-4.30
Fv/Fm	Altay2000	0.73	0.67	8.22
	UZ-11CWA-8	0.72	0.71	1.39
	Bobur	0.73	0.74	-1.37
	P1G082	0.74	0.73	1.35
	P1G119	0.77	0.73	5.19
	P1G202	0.72	0.71	1.39
	P1G264	0.75	0.73	2.67
	P1G132	0.72	0.73	-1.39
	P1G253	0.71	0.73	-2.82
	P2G076	0.74	0.73	1.35
	P2G243	0.73	0.72	1.37
	P2G027	0.72	0.73	-1.39
	P2G162	0.74	0.75	-1.35

Fm/Fo, non-photochemical loss in PSII; Fv/Fo, efficiency of the water splitting complex; Fv/Fm, maximum quantum yield of PSII.

Table S8. Candidate genes for the significant marker–trait associations for salinity tolerance in the contrasting wheat genotypes. Their functions were adapted from the JBrowse (https://urgi.versailles.inra.fr/jbrowseiwgsc/gmod_jbrowse/?data=myData%2FIWGSC_RefSeq_v1.0&loc=chr3A%3A515889718..515889909&) database.

Gene ID	Genes Number	Chr.	Gene Position(bp)	Gene Length(bp)	Gene Annotation
TraesCS1D02G052200	Gene_1	1DS	33469805..33474180	4,376	nucleotide binding (GO:0000166)<br>alcohol dehydrogenase (NAD+) activity (GO:0004022)<br>catabolic process (GO:0009056)<br>zinc ion binding (GO:0008270)<br>response to salt stress (GO:0009651)
TraesCS1D02G052700	Gene_2	1DS	33638501..33640761	2,261	leaf senescence (GO:0010150)<br>abscisic acid stimulus (GO:0071215)<br>cellular response to salicylic acid stimulus (GO:0071446)<br>cellular response to osmotic stress (GO:0071470)
TraesCS1D02G054400	Gene_3	1DS	35788030..35788783	754	response to salt stress (GO:0009651)
TraesCS1D02G054500	Gene_4	1DS	35886292..35900512	14,221	response to salt stress (GO:0009651)
TraesCS1D02G054600	Gene_5	1DS	35926381..35927231	851	response to salt stress (GO:0009651)
TraesCS2B02G503100	Gene_6	2BS	697546485..697548362	1,878	response to toxic substance (GO:0009636)<br>response to salt stress (GO:0009651)
TraesCS5B02G368500	Gene_7	5BL	546826331..546832103	5,773	potassium ion transmembrane transport (GO:0071805)<br>potassium ion transmembrane transporter activity (GO:0015079)
TraesCS5B02G368800	Gene_8	5BL	547399295..547406590	7,296	sodium ion transport (GO:0006814)<br>chloride ion homeostasis (GO:0055064)<br>potassium ion homeostasis (GO:0055075)<br>potassium ion transmembrane transport (GO:0071805)<br>chloride transmembrane transport (GO:1902476)<br>sodium:potassium:chloride symporter activity (GO:0008511)<br>potassium:chloride symporter activity (GO:0015379)
